# Genetic Traces of the *Francisella tularensis* Colonization of Spain, 1998–2020

**DOI:** 10.3390/microorganisms8111784

**Published:** 2020-11-14

**Authors:** Kerstin Myrtennäs, Raquel Escudero, Ángel Zaballos, Rosa González-Martín-Niño, Miklós Gyuranecz, Anders Johansson

**Affiliations:** 1 Division of CBRN Defence and Security, Swedish Defence Research Agency (FOI), 90182 Umeå, Sweden; 2 Instituto de Salud Carlos III, Centro Nacional de Microbiología, 28220 Madrid, Spain; rescude@isciii.es (R.E.); azaballos@isciii.es (Á.Z.); rmgonzalez@isciii.es (R.G.-M.-N.); 3 Institute for Veterinary Medical Research, Centre for Agricultural Research, Hungária körút 21, 1143 Budapest, Hungary; m.gyuranecz@gmail.com; 4 Department of Clinical Microbiology, Umeå University, SE-901 87 Umeå, Sweden; anders.f.johansson@umu.se

**Keywords:** Spain, tularemia, *Francisella*, SNP, VNTR, NGS, sequencing, colonization, evolution, epidemiology

## Abstract

More than 1000 humans have acquired the febrile disease tularemia in Spain since the first notification of human cases in 1997. We here aimed to study the recent molecular evolution of the causative bacterium *Francisella tularensis* during disease establishment in Spain. Single-nucleotide polymorphisms (SNPs) and variable-number tandem repeats (VNTRs) were analyzed in whole-genome sequences (WGS) of *F. tularensis*. Short-read WGS data for 20 *F. tularensis* strains from humans infected in the periods 2014–2015 and 2018–2020 in Spain were generated. These data were combined with WGS data of 25 Spanish strains from 1998 to 2008 and two reference strains. Capillary electrophoresis data of VNTR genetic regions were generated and compared with the WGS data for the 11 strains from 2014 to 2015. Evolutionary relationships among strains were analyzed by phylogenetic methods. We identified 117 informative SNPs in a 1,577,289-nucleotide WGS alignment of 47 *F. tularensis* genomes. Forty-five strains from Spain formed a star-like SNP phylogeny with six branches emerging from a basal common node. The most recently evolved genomes formed four additional star-like structures that were derived from four branches of the basal common node. VNTR copy number variation was detected in two out of 10 VNTR regions examined. Genetic clustering of strains by VNTRs agreed with the clustering by SNPs. The SNP data provided higher resolution among strains than the VNTRs data in all but one cases. There was an excellent correlation between VNTR marker sizing by capillary electrophoresis and prediction from WGS data. The genetic data strongly support that tularemia, indeed, emerged recently in Spain. Distinct genetic patterns of local *F. tularensis* population expansions imply that the pathogen has colonized a previously disease-free geographical area. We also found that genome-wide SNPs provide higher genetic resolution among *F. tularensis* genomes than the use of VNTRs, and that VNTR copy numbers can be accurately predicted using short-read WGS data.

## 1. Introduction

*Francisella tularensis* causes the zoonotic disease tularemia. In Europe, the *F. tularensis* subspecies *holarctica* is the causative agent often resulting in a febrile, long-lasting disease characterized by lymph node enlargement among humans (type B tularemia) but with a rate of mortality less than one percent. In North America, type B tularemia exists side by side with type A tularemia, a disease that is caused by the *F. tularensis* subspecies *tularensis*, with a mortality among humans of several percent. Infected insect or tick bites or direct contact with sick small mammals are common routes of infection for humans in both types of tularemia [1]. It appears that *F. tularensis* can persist locally somewhere in the environment between seasonal outbreaks of disease [2,3,4,5,6]. The advancement of genetic typing methods for *F. tularensis* has shown that the evolution of *F. tularensis* is clonal, that the subspecies *holarctica* evolved recently, and that this subspecies has successfully spread across large geographical distances in Europe [7,8,9,10,11,12]. The limited genetic variation and the clonal inheritance pattern of this bacterium are helpful properties in the interpretation of the genetic kinship of *F. tularensis*. Commonly used genetic markers for evolutionary and epidemiology studies of *F. tularensis* include multi-locus variable-number tandem repeats (VNTRs) [13,14,15] and single-nucleotide polymorphisms (SNPs) mapped in a few genes or at the whole genome scale [7,8,9,10,11,12]. It has been argued that markers with a more rapid evolution, such as VNTRs, could be especially useful for tracking the recent evolution of *F. tularensis* [13], but it is unknown if this property of VNTRs provides as much discrimination as the more recent approach of mapping SNPs in the full genome of the bacterium using whole-genome sequencing (WGS) [14,16].

In Spain, tularemia was first reported in 1997 when an outbreak affected 585 humans [17]. More than 1000 patients have since been reported, with a geographical focus of disease in the autonomous community of Castilla y León. All the infections in humans and animals in Spain appear to be caused by a single, genetically extremely-coherent subpopulation of *F. tularensis* subspecies *holarctica* [16]. This situation with intense disease activity and a recent disease introduction should provide an excellent opportunity for investigating bacterial evolution in a natural environment. Bacteria cultured from infected individuals in recurrent outbreaks in Spain can provide evolutionary snap-shots of an *F. tularensis* population of very recent common ancestry.

The choice of genetic markers for evolutionary and epidemiology studies of *F. tularensis* has, to some extent, been influenced by the technology available for mapping the markers. VNTRs were popular markers when capillary electrophoresis for detecting VNTR copy-number variation became widely available in the late 1990s [14,18]. A few years later, when short-read WGS, which was ideal to reliably map SNPs, became cheaper and widely available, VNTRs were more or less abandoned [8].

Here, we describe the recent molecular evolution of *F. tularensis* in Spain by utilizing short-read WGS data. We have investigated the relationship between two types of genetic markers, VNTRs and SNPs, in these data. In addition, we investigated the reliability of predicting VNTR copy numbers from WGS data by comparison with capillary electrophoresis data.

## 2. Materials and Methods

### 2.1. Strains

Twenty *F. tularensis* strains isolated from humans in 2014–2015 and 2018–2020 and 25 strains isolated in 1998 and 2007–2008 in the autonomous community of Castila y León, Spain, were included (Table 1). The latter 25 strains were previously genome-sequenced [16] (summarized in Appendix A Table A1). In addition, reference strains from Italy (FSC031; O-407) and France (FTNF002-00 (FTA) GenBank acc. no. CP000803.1.) were included.

### 2.2. Genome Sequencing

The 20 strains isolated in 2014–2015 and 2018–2020 were cultivated on cysteine heart agar supplemented with 9% chocolatized red blood cells (CHAB). DNA was prepared and purified using the QIAamp DNA mini kit (Qiagen, Hilden, Germany). A library was prepared using the Nextera XT kit and sequenced at the Centro Nacional de Microbiología-Instituto de Salud Carlos III, Genomic Unit, on a MiSeq instrument (Illumina Inc., San Diego, CA, USA), which produced 2 × 300 bp pair-end read data. The genomes were assembled using ABySS 1.5.1 [19] and submitted to GenBank under BioProject accession number PRJNA548692.

### 2.3. Taxonomic Classification

Each genome was assigned to a genetic clade using CanSNPer [20,21]. Upon detection of a new clade with at least two taxa, this clade was given a new taxonomic canSNP index following the scheme at Github [20]. One SNP per branch was selected to define the whole branch.

### 2.4. Sizing of PCR Products Targeting VNTRs with Capillary Electrophoresis and Genome Sequencing

PCR product capillary electrophoretic sizing, or fragment analysis for short, of ten VNTRs (Ft-M2, Ft-M3, Ft-M4, Ft-M5, Ft-M6, Ft-M10, Ft-M20, Ft-M22, Ft-M23, and Ft-M24) was performed at the Institute for Veterinary Medical Research, Hungarian Academy of Science, on an ABI Prism 3100 Genetic Analyzer (Applied Biosystems, Foster City, CA, USA) using primers described in Appendix A Table A2, and under the following conditions: the PCRs were done in single-plex in a 25-μL volume using 0.5–2 μL of each primer, 1 μL DNA template, and a reaction mixture with 5 μL 5× Colorless GoTaq Flexi Buffer (Thermo Fisher Scientific, Waltham, MA, USA), 2 μL 25 mM MgCl_2_ (Thermo Fisher Scientific), 0.8 μL 10 mM dNTP (Thermo Fisher Scientific), and 0.2–0.4 μL GoTaq Polymerase (5 unit/μL) (Thermo Fisher Scientific). The cycling conditions were 94 °C for 5 min and then 35 cycles with denaturation at 94 °C for 30 s, primer annealing at 58 °C for 30 s, and extension at 72 °C for 30 s. The program ended with a final extension at 72 °C for 30 s [21]. The PCR product size was determined with Peak ScannerTM Software v.1.0 (Applied Biosystems). These ten markers have previously been used to subtype the subspecies *holarctica* from Europe [13]. The marker Ft-M20 was targeted using two assays—Ft-M20A and Ft-M20B.

Sizing of PCR products was performed using an in-house Python script by mapping the primer sequences to each genome.

### 2.5. Phylogenetics and Phylogeography

Single-nucleotide polymorphisms and genetic distances among the strains were determined using a whole-genome alignment produced by progressiveMauve with default settings. A neighbor-joining tree based on the core of the 47 genomes was constructed in MEGAX (Tamura et al.) with the number of differences method and complete deletion. The SNP phylogeny was compared with VNTR marker patterns. The geographical and temporal distributions of *F. tularensis* strains belonging in different phylogenetic clades were analyzed for the seven provinces of Spain experiencing tularemia outbreaks.

## 3. Results

### 3.1. Genome Sequencing, Assembly and SNP Identification

**Figure 1:**
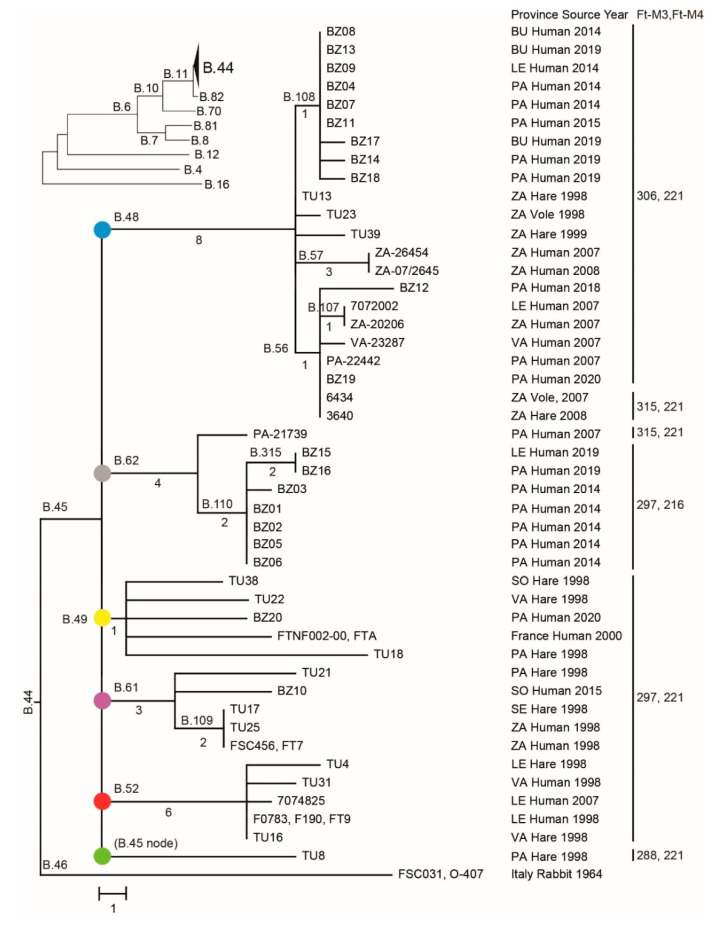
Neighbor-joining phylogeny based on 117 single-nucleotide polymorphisms (SNPs) in whole-genome sequencing (WGS) data of 45 *F. tularensis* strains from Spain, 1998 to 2020, and two reference genomes from France and Italy demonstrating a star-like branching pattern. The tree is rooted in the Italian genome as this genome is the most ancestral in relation to the global *Francisella tularensis* subspecies *holarctica* phylogeny (see the upper-left inset tree). Major genetic groups of strains from Spain are labelled by color-filled circles. Tree branches are labelled by canSNPs (the letter B followed by a number). The number of SNPs along the branches is indicated for branches with multiple taxa (see Appendix A Table A4 for canSNP positions in the reference strain FTNF002-00). Province abbreviations are Burgos (BU), León (LE), Palencia (PA), Segovia (SE), Soria (SO), Valladolid (VA), and Zamora (ZA). Variable-number tandem repeat (VNTR) marker data are in the right column.

We performed short-read sequencing and created new genome assemblies for 20 *F. tularensis* isolates cultured from diagnostic specimens of humans infected in Spain in 2014–2015 and 2018–2020. The assemblies typically consisted of 100 contigs with ends corresponding to the multiple copy insertion sequence elements ISFtu1 or ISFtu2 or the multiple copy ribosomal operon of *F. tularensis* (Appendix A, Table A3). The mean sequence depth (genome coverage) ranged from 89 to 578. These new sequence data were analyzed together with previously published genome sequence assemblies of 25 *F. tularensis* genomes from Spain using a common analysis pipeline. This resulted in a core genome of 1,577,289 nt, including genes and intergenic regions excluding multiple copy insertion sequence elements and ribosomal operons representing 45 genomes from Spain and two closely related reference genomes from Italy and France. The core genome size corresponds to 88% of the total reference genome of the strain SCHU S4, including its multiple copy insertion sequence elements and ribosomal operons. We found 117 SNPs in the core genome of the 47 genomes analyzed in this work (Figure 1), corresponding to a core genome identity of 99.9925%.

### 3.2. Taxonomic Classification

To facilitate future comparisons with genomes included in this study, all genomes were assigned to the general taxonomic system built on canSNPs of *F. tularensis*. To this end, 34 SNPs defined 13 genetic clades having more than one member among the 47 strains, i.e., these canSNPs were not strain-specific (Appendix A, Table A4). Five of the 13 genetic clades were new to this study and the four branches leading to these new clades were indexed from B.107 to B.110 and B.315. One canonical SNP per new branch was selected to represent each branch as follows: B.107 (canSNP 520,520), B.108 (1,327,297), B.109 (1,082,513), B.110 (1,301,561), and B.315 (1,721,277), with the positions in brackets referring to the genome FTNF002-00 (FTA) with GenBank acc. no. CP000803.1. The canSNP typing scheme is available at Github [20], including the five new canSNPs described in this work.

### 3.3. Analysis of VNTRs by Short Read Genome Sequencing and Capillary Electrophoresis

**Figure 2:**
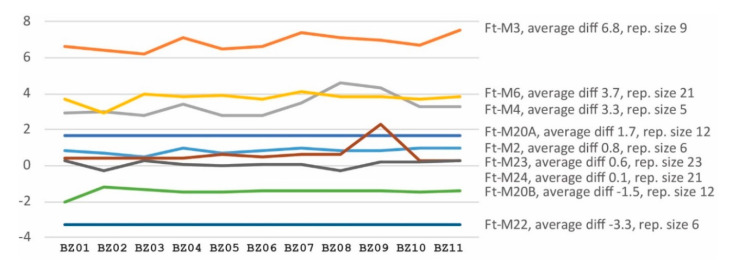
Difference between VNTR PCR product sizes determined by capillary electrophoresis and by WGS data. Eleven *F. tularensis* strains from 2014 to 2015 are named along the *X*-axis. Dissimilarities in the size estimates (no. of nucleotides) are shown on the *Y*-axis. The Ft-M5 and Ft-M10 data are incomplete because these VNTR loci are adjacent to a large insertions-sequence element at the end of contigs in the WGS data.

To investigate if VNTR copy numbers in *F. tularensis* genomes can be predicted using short-read genome sequence data, we compared PCR product capillary electrophoretic VNTR sizing versus sizes from short-read data. We found that only two VNTR markers out of the ten VNTR markers tested by capillary electrophoresis varied between the 47 *F. tularensis* strains (Appendix A, Table A5 and Table A6). Data for the two variable markers, that are named Ft-M3 and Ft-M4, can be found in Table 2 for the 11 genomes with WGS data generated in this study. No variation was observed for any other marker among the 47 strains (Ft-M2, Ft-M5, Ft-M6, Ft-M10, Ft-M20A, Ft-M20B, Ft-M22, Ft-M23, and Ft-M24 were invariable). Comparison of VNTR sizes predicted from capillary electrophoresis fragment analysis versus WGS data revealed that the size estimates were consistent across different genomes (Figure 2 and Appendix A Table A7). The analysis showed that translation from VNTR sizes obtained by fragment analysis to true VNTR sizes in the WGS data needs marker-specific correction of the former, and that this is especially important for markers with a short repeat size (<10 bp).

### 3.4. Phylogenetic Analysis Using SNP or VNTR Data

The phylogenetic tree based on 117 core SNPs displayed a star-like shape with multiple hard polytomies, of which the most basal polytomy had six tree branches corresponding to genetic distances ranging from 1 to 8 nt, emerging from a common node representing the most recent common ancestor (Figure 1). Four additional polytomies were present among the downstream branching patterns of the tree. The SNP inheritance pattern along the different branching structures of the tree was strictly clonal, i.e., there were no SNPs that were inconsistent with the tree structure. The genetic clustering by VNTR markers agreed to the clustering by SNPs, although the VNTR markers in all but one cases resulted in a lower resolution among taxa (Figure 1, strains 3640 and 6434). There were, in total, 23 SNP genotypes that matched six VNTR genotypes.

### 3.5. Phylogeography

**Figure 3:**
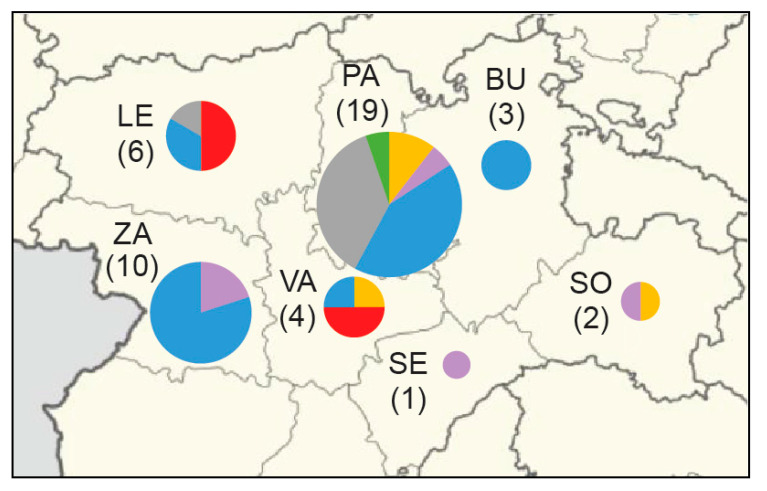
Phylogeography of 45 *F. tularensis* strains with a known place of isolation in seven Spanish provinces from 1998 to 2020. Major phylogenetic clades are labelled by colors corresponding to Figure 1. Province labels are Burgos (BU), León (LE), Palencia (PA), Segovia (SE), Soria (SO), Valladolid (VA), and Zamora (ZA). The number of genomes is within round brackets.

**Figure 4:**
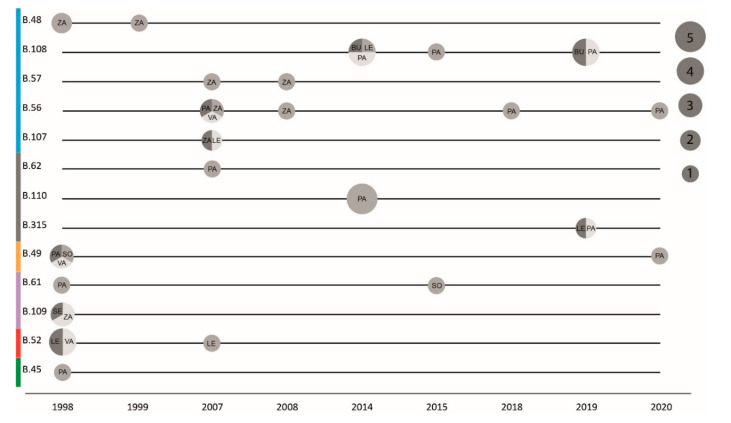
Timing and size of *F. tularensis* genetic clades. Horizontal lines represent the different phylogenetic clades over time. The circle size represents the number of genomes, with grey shades distinguishing different provinces. The major phylogenetic clades are color-coded on the *Y*-axis, corresponding to Figure 1. Provinces are Burgos (BU), León (LE), Palencia (PA), Segovia (SE), Soria (SO), Valladolid (VA), and Zamora (ZA).

The phylogeography of six major clades including 45 *F. tularensis* strains with a known place of isolation in seven Spanish provinces is shown in Figure 3. The total diversity at the clade level and their spatiotemporal distribution is illustrated in Figure 1 and Figure 4. Most strains and the largest genetic diversity were from the province of Palencia. We found that *F. tularensis* strains belonging to two distinct clades, B.108 and B.110, caused the 2014–2015 human tularemia outbreaks in Palencia. Notably, we found that a single strain ancestral to B.110, named PA-21739, was isolated from a human in Palencia as early as 2007. In 2018–2019, strains of a new subclade, B.315, which is closely related to B.110, caused new outbreaks in Palencia. In contrast to this regional pattern of genetic homogeneity over time, Palencia strains from 2020 were genetically more diverse, belonging in B.49 and B.56. The diversity was apparently present in Palencia already in 1998, as we found single isolates obtained from hares in three diverse major clades, B.49, B.61, and B.45. The largest genetic distance within a clade with strains from Spain was 15 SNPs and was found between two strains from Palencia in 1998 and 2020 (clade B.49). The human epidemiological data connected to the 20 *F. tularensis* strains from 2014–2015 and 2018–2020 suggested that the disease had been acquired during outdoor activities (Table 1).

## 4. Discussion

The introduction of tularemia into Spain offered an opportunity to study the recent molecular evolution of *F. tularensis* in relation to disease emergence in a new geographical area. We found an evolutionary pattern strongly suggesting recent pathogen population expansion in Spain. The data are consistent with epidemiological records of recent disease import into Spain and show that the *F. tularensis* population size has rapidly increased locally, now causing disease among large numbers of humans and small mammals. We also found that two types of evolutionary marker in the *F. tularensis* genomes, SNPs and VNTRs, essentially tell the same story.

The analysis of 20 new *F. tularensis* genomes from 2014 to 2015 and 2018 to 2020 and 25 older genomes of strains from 1998 and 2007–2008, revealed a star-like phylogeny with six branches emerging from a common basal node, a pattern supporting a recent pathogen population expansion. A basal star-like structure was accompanied by additional star-like branching structures further out on the tree branches signifying that the initial population expansion was followed by additional population expansions within the country. We previously reported a star-like phylogeny for *F. tularensis* in an investigation of disease introduction from more Eastern countries into Western Europe as a whole, but it was not as pronounced as observed here [16]. Other recent reports of the migration pattern of *F. tularensis* in Europe include studies from France, Switzerland, Germany, and Austria [22,23,24,25]. The star-like phylogenetic pattern seen here is an example of rapidly increasing biodiversity at the subspecies level of a genetically monomorphic infectious agent. The phenomenon as such, however, is more often described at a higher taxonomic level in eukaryotic species and is named evolutionary radiation, one of the examples being Darwin’s finches. In the current work, we have captured bacterial subpopulations that are in very early stages of increasing biodiversity connoted by star-like structures in analyses of bacterial WGS data. Evolutionary theory predicts that the number of star-like structures (evolutionary radiations) will be reduced with evolutionary time, as genetic variants within an evolutionary radiation become extinct by random and selection effects; see Simões et al. for a review of the current understanding of genetic radiation patterns [26]. Indeed, star-like structures appear to get lost over time in *F. tularensis*. In countries with long-term tularemia endemicity, e.g., in Sweden, Norway, or Turkey [7,11,12], having large disease outbreaks since many decades, *F. tularensis* WGS star phylogenies are rare and found at the tree tips only. In these countries, phylogenies are characterized by long tree branches between major genetic groups of strains, each major group having many unique genotypes. In line with evolutionary predictions, these long tree branches represent the few bacterial genetic lineages that did not become extinct during long-term evolution. A recent genome comparison of 350 *F. tularensis* strains from France, where tularemia emerged between 1940 and 1950, provides additional support for this scenario with star-like phylogeny formation followed by random extinction of genetic variants over longer time frames [25]. The comprehensive analysis of *F. tularensis* genomes from France showed the emergence of longer tree branches separating star-like structures at the tip of the phylogenetic tree. The stars at the tree-tips among the genomes from France generally represented strains of recent origin, isolated after 1990. Taken together, these earlier findings from Sweden, Norway, Turkey, and France coupled to the present findings from Spain support that WGS analyses of *F. tularensis* provide some information about how long tularemia has been endemic in a particular geographical area.

A probable explanation of the rapid expansion of *F. tularensis* populations in Castilla-y-León, Spain, is that human activities have created excellent opportunities for pathogen expansion. The *F. tularensis* subspecies *holarctica* is known to be associated with water [27], and common disease transmission paths to humans include vector bites, crayfish fishing, and contact with infected mammals [27,28,29]. The natural climate of Castilla-y-León, however, is hostile to *F. tularensis* subspecies *holarctica,* with hot and dry summers and mesic habitats that restrict the abundance of ticks. To solve the problem of agricultural drought, an extensive network of irrigation water canals and ditches have been established from the 1970s to 1990s. This solution, at the same time, provides an excellent habitat for *F. tularensis* subspecies *holarctica* to survive and replicate [30]. The irrigation system has caused an explosion of voles in the irrigated fields. As voles easily get tularemia, these animals can serve to amplify the expansion of *F. tularensis* populations. The beginning of the tularemia outbreak in Spain in 1997 coincides with this change in the local ecology. Additional sampling in irrigation channels, rivers, and lagoons and animals in 2016–2017 has confirmed the presence of *F. tularensis* in water, sediments, hares, and ticks in the local area (personal communications, M Dolores Vidal, Instituto de Salud Carlos III, Madrid, Spain).

We also wanted to investigate if WGS data are suitable for predicting VNTR copy numbers in *F. tularensis* because these markers are known to evolve fast and may complement SNPs as markers of recent evolution. Our results show that capillary electrophoresis sizing is not any longer necessary for obtaining VNTR data in *F. tularensis*. Both SNP and VNTR data can be extracted from the same WGS short reads, meaning that draft sequencing provides a simpler, faster, and cheaper method for VNTR typing. Our work further illustrates the need for marker-specific correction when using capillary electrophoresis sizing of VNTRs, which, if not performed, complicates comparison between studies. Finally, we found, contradictory to the assumption that VNTRs evolve faster than SNPs and, therefore, should be better for resolving recent evolution, that the use of SNPs across the whole *F. tularensis* genome gave a higher typing resolution than VNTRs in all but one cases. One simple explanation for the observation is that SNPs across the genomes occur in higher numbers, i.e., 117 different SNPs compared with 10 VNTR regions.

## 5. Conclusions

The phylogenetic analysis strongly supported a recent rapid expansion of *F. tularensis* populations in Spain, consistent with the recent disease introduction into the country and that human activities may have facilitated this expansion. The use of whole-genome SNPs provided higher resolution than the use of multiple VNTRs for depicting *F. tularensis* evolution. We found that VNTR copy numbers of *F. tularensis* can be accurately predicted using short-read WGS data but provide little extra information for epidemiological studies.

## Figures and Tables

**Table 1:** *Francisella tularensis* strains from 20 human specimens in Spain, 2014–2020.

ID	Source	Transmission Related to	Year	Province
BZ01	Skin ulcer	Crayfish	2014	Palencia
BZ02	Skin ulcer	Crayfish	2014	Palencia
BZ03	Skin ulcer	Crayfish/rodents/gardening	2014	Palencia
BZ04	Skin ulcer	Crayfish/countryside walks	2014	Palencia
BZ05	Blood	Rodent/agriculture	2014	Palencia
BZ06	Skin ulcer	Crayfish	2014	Palencia
BZ07	Blood	Country house	2014	Palencia
BZ08	Transbronchial needle aspiration of lymph node	Environment	2014	Burgos
BZ09	Skin ulcer	Hunting	2014	Leon
BZ10	Skin ulcer	Hunting	2015	Soria
BZ11	Blood	Not available	2015	Palencia
BZ12	Blood	Farming	2018	Palencia
BZ13	Skin abscess	Crayfish	2019	Burgos
BZ14	Blood	Gardening	2019	Palencia
BZ15	Lymph node abscess	Not available	2019	León
BZ16	Lymph node abscess	Crayfish	2019	Palencia
BZ17	Skin ulcer	Crayfish	2019	Burgos
BZ18	Skin ulcer	Hunting	2019	Palencia
BZ19	Blood	Not available	2020	Palencia
BZ20	Lymph node abscess	Countryside walks	2020	Palencia

**Table 2:** Capillary electrophoresis sizing of VNTR markers Ft-M3 and Ft-M4 in relation to nucleotide size data determined by WGS of 11 *F. tularensis* genomes from 2014 to 2015, Spain.

	Ft-M3 (9 bp Repeat Size)			Ft-M4 (5 bp Repeat Size)		
ID	No. Repeats	Assay	WGS	No. Repeats	Assay	WGS
BZ01	8	303.6	297	1	218.9–219.9	216
BZ02	8	303.4	297	1	219.0	216
BZ03	8	303.2	297	1	218.8–219.8	216
BZ04	9	313.1	306	2	224.4	221
BZ05	8	303.5	297	1	218.8–219.8	216
BZ06	8	303.6	297	1	218.8–219.8	216
BZ07	9	313.4	306	2	224.5	221
BZ08	9	313.1	306	2	225.6	221
BZ09	9	313.0	306	2	225.3	221
BZ10	8	303.7	297	2	224.3	221
BZ11	9	313.5	306	2	224.3	221

## Appendix A

**Table A1:** Twenty-five strains isolated in 1998 and 2007–2008 in the autonomous community of Castilla y León, Spain.

ID	Source	Province	Year of Isolation	Accession ^1^	Clade
F0783, FT9, F190	Human	León	1998	SAMN03773734	B.52
TU4	Hare	León	1998	SAMN03773884	B.52
TU21	Hare	Palencia	1998	SAMN03773885	B.61
TU39	Hare	Zamora	1999	SAMN03773886	B.48
TU8	Hare	Palencia	1998	SAMN03773887	B.45
TU17	Hare	Segovia	1998	SAMN03773888	B.61
TU18	Hare	Palencia	1998	SAMN03773889	B.49
TU16	Hare	Valladolid	1998	SAMN03773890	B.52
TU38	Hare	Soria	1998	SAMN03773891	B.49
TU23	Vole	Zamora	1998	SAMN03773892	B.48
TU22	Hare	Valladolid	1998	SAMN03773893	B.49
TU31	Human	Valladolid	1998	SAMN03773894	B.52
TU13	Hare	Zamora	1998	SAMN03773895	B.48
TU25	Human	Zamora	1998	SAMN03773896	B.61
VA-23287	Human	Valladolid	2007	SAMN03773897	B.56
ZA-07/2645	Human	Zamora	2008	SAMN03773898	B.57
7072002	Human	León	2007	SAMN03773899	B.56
6434	Vole	Zamora	2007	SAMN03773900	B.56
3640	Hare	Zamora	2008	SAMN03773901	B.56
ZA-26454	Human	Zamora	2007	SAMN03773902	B.57
PA-21739	Human	Palencia	2007	SAMN03773903	B.62
ZA-20206	Human	Zamora	2007	SAMN03773904	B.56
7074825	Human	León	2007	SAMN03773905	B.52
PA-22442	Human	Palencia	2007	SAMN03773906	B.56
FSC456, FT7	Human	Zamora	1998	SAMN03774111	B.61

^1^ Dwibedi et al., [16].

**Table A2:** Primers for PCR product sizing using capillary electrophoresis.

Primer	Sequence	Fluorescence Dye	Melting Point
Ft-M03_F	gcacgcttgtctcctatcatcctctggtagtc	HEX	64.7 °C
Ft-M03_R	gaacaacaaaagcaacagcaaaattcacaaaa		59.4 °C
Ft-M04_F	gcgcgctatctaactaatttttatattgaaacaatcaaat	FAM	59.3 °C
Ft-M04_R	gcaaatataccgtaatgccacctatgaaaactc		60.2 °C
Ft-M05_F	gtttgttacgccaataaacaaaaagtgtaaataatg	NED	57.6 °C
Ft-M05_R	gctcagctcgaactccgtcataccttcttc		64.1 °C
Ft-M10_F	gctaattttttcatatttatctccatttttacttttttgc	HEX	57 °C
Ft-M10_R	gctcagctcgaactccgtcataccttcttc		64.1 °C
Ft-M20A_F	gtatatcttggaataagccggagttagatggttct	FAM	61.1 °C
FtM20A_R	gcaataactttatcacccttattgtagactgcttctgc		62.1 °C
Ft-M20B_F	gggtgataaagttattgttaatggtgtgacttatgaa		59.5 °C
Ft-M20B_R	gtaactacttgaccgccagtatatgcttgacct	HEX	63 °C
Ft-M06_F	gtttttggtgaactgccaacaccataactt	NED	61.1 °C
Ft-M06_R	gcaattcagcgaaaccctatcttagcctc		62 °C
Ft-M02_F	gctgctgtggctgtaaatgttgctatgct	FAM	64.1 °C
Ft-M02_R	gcagggcacaattcttgaccatcagg		63.3 °C
Ft-M22_F	gtggaaatgcaaaaacaatatcgaggaacttta	FAM	59.1 °C
Ft-M22_R	gttttttctcgtccgctgttagtgattttacatc		60.5 °C
Ft-M23_F	gctggattattaaagcatatgacagacgagtagg	NED	60.6 °C
Ft-M23_R	gttccctcaggtttatccaaagttttaatgttttatt		59 °C
Ft-M24_F	gaatcttaatccatacggtcctaataatattcctgtcaat	NED	59.8 °C
Ft-M24_R	gttggtacttatgggctatagcggatattatttcagt		61.2 °C

**Table A3:** Genome information for 20 new genomes from Spain, 2014–2015 and 2018–2020.

ID	Total No. Bases	Total No. Contigs	N50	Mean Sequence Depth	BioSample Accession *	Clade	Subclade Path
BZ01	1,810,591	95	27699	455	SAMN12071621	B.62	B.62–B.110
BZ02	1,821,580	95	27699	431	SAMN12071622	B.62	B.62–B.110
BZ03	1,825,760	111	23863	466	SAMN12071623	B.62	B.62–B.110
BZ04	1,823,438	98	26961	481	SAMN12071624	B.48	B.48–B.108
BZ05	1,809,217	97	25525	410	SAMN12071625	B.62	B.62–B.110
BZ06	1,825,942	105	25544	574	SAMN12071626	B.62	B.62–B.110
BZ07	1,812,133	98	26732	550	SAMN12071627	B.48	B.48–B.108
BZ08	1,818,495	94	27699	559	SAMN12071628	B.48	B.48–B.108
BZ09	1,825,893	108	25990	424	SAMN12071629	B.48	B.48–B.108
BZ10	1,811,236	88	33271	417	SAMN12071630	B.61	B.61
BZ11	1,808,131	92	29735	514	SAMN12071631	B.48	B.48–B.108
BZ12	1,830,400	100	26972	340	SAMN16515973	B.48	B.48–B.56
BZ13	1,756,606	218	15177	269	SAMN16515974	B.48	B.48–B.108
BZ14	1,775,418	201	15465	282	SAMN16515975	B.48	B.48–B.108
BZ15	1,818,738	99	26747	242	SAMN16515976	B.62	B.62–B.110–B.315
BZ16	1,813,941	97	26857	281	SAMN16515977	B.62	B.62–B.110–B.315
BZ17	1,819,785	97	27089	331	SAMN16515978	B.48	B.48–B.108
BZ18	1,708,033	369	7369	231	SAMN16515979	B.48	B.48–B.108
BZ19	1,833,295	94	27956	103	SAMN16515980	B.48	B.48–B.56
BZ20	1,834,431	89	29599	89	SAMN16515981	B.49	B.49

* BioProject no. PRJNA548692.

**Table A4:** Thirty-four identified canonical SNPs for clades in Figure 1 with more than two members. The clades are in the same order as they appear in Figure 1. The position in the reference genome of FTNF002-00 (FTA) is indicated. Members of clades are shown. The canonical SNPs selected to represent the branches for five new clades, B.107–B.110 and B.315, are indicated in bold.

Clade	Genomes	No. SNPs	SNP Position (Anc/Der)
B.48	BZ04:BZ07:BZ08:BZ09:BZ11: BZ12:BZ13:BZ14:BZ17:BZ18: BZ19:TU39:TU23:TU13: VA-23287:ZA-07/2645:7072002: 6434:3640:ZA-26454: ZA-20206:PA-22442	8	170,704 (C/T);258,998 (G/T); 736,278 (G/A);1,075,715 (C/T); 1,194,117 (G/A);1,225,490 (G/A); 1,558,703 (C/T);1,746,518 (C/T)
B.108	BZ04:BZ07:BZ08:BZ09:BZ11: BZ13:BZ14:BZ17:BZ18	1	**1,327,297 (C/T)**
B.57	ZA-07/2645:ZA-26454	3	668,630 (T/C);1,747,062 (G/T);1,860,651 (G/A)
B.56	BZ12:BZ19:VA-23287: 7072002:6434:3640:ZA-20206: PA-22442	1	827,994 (G/A)
B.107	7072002:ZA-20206	1	**520,520 (C/T)**
B.62	BZ01:BZ02:BZ03:BZ05:BZ06: BZ15:BZ16:PA-21739	4	155,417 (G/A);411,903 (C/T); 505,602 (C/T);691,772 (C/T)
B.110	BZ01:BZ02:BZ03:BZ05:BZ06: BZ15:BZ16	2	**1,301,561 (G/A)**;1,742,563 (G/A)
B.315	BZ15:BZ16	2	975,621 (G/C);**1,721,277 (T/C)**
B.61	BZ10:TU21:TU17:TU25:FSC456	3	235,472 (G/A);1,009,840 (G/T);1,332,119 (G/A)
B.109	TU17:TU25:FSC456	2	**1,082,513 (T/C)**;1,171,726 (G/A)
B.49	BZ20:TU18:TU38:TU22:FTNF002	1	224,367 (G/A)
B.52	F0783:TU4:TU16:TU31:7074825	6	238,894 (C/T);324,043 (C/T); 1,009,935 (C/T);1,130,906 (C/T); 1,151,666 (T/C);444,395 (C/T)

**Table A5:** Sizes of PCR Fragments targeting ten VNTR markers determined by capillary electrophoresis.

ID	Ft-M2	Ft-M3	Ft-M4	Ft-M5	Ft-M6	Ft-M10	Ft-M20A	Ft-M20B	Ft-M22	Ft-M23	Ft-M24
BZ01	339.8	303.6	218.9–219.9	299.6	275.7	199.6	307.7	151.0	174.7	327.4	400.3
BZ02	339.7	303.4	219.0	300.0	274.9	199.6	307.7	151.8	174.7	327.4	399.7
BZ03	339.5	303.2	218.8–219.8	300.3	276.0	199.6	307.7	151.7	174.7	327.4	400.3
BZ04	340.0	313.1	224.4	300.3	275.8	199.6	307.7	151.5	174.7	327.4	400.1
BZ05	339.7	303.5	218.8–219.8	300.0	275.9	199.6	307.7	151.5	174.7	327.6	400.0
BZ06	339.8	303.6	218.8–219.8	300.1	275.7	199.6	307.7	151.6	174.7	327.5	400.1
BZ07	340.0	313.4	224.5	300.3	276.1	199.6	307.7	151.6	174.7	327.6	400.1
BZ08	339.8	313.1	225.6	299.7	275.8	199.6	307.7	151.6	174.7	327.6	399.7
BZ09	339.8	313.0	225.3	300.2	275.8	199.6	307.7	151.6	174.7	327.3	400.2
BZ10	340.0	303.7	224.3	300.2	275.7	199.6	307.7	151.5	174.7	327.3	400.2
BZ11	340.0	313.5	224.3	300.1	275.8	199.6	307.7	151.6	174.7	327.3	400.3

**Table A6:** Sizing of PCR fragments targeting ten VNTR markers from genomes.

ID	Ft-M2	Ft-M3	Ft-M4	Ft-M5	Ft-M6	Ft-M10	Ft-M20A	Ft-M20B	Ft-M22	Ft-M23	Ft-M24
BZ01	339	297	216	-	272	-	306	153	178	327	400
BZ02	339	297	216	-	272	-	306	153	178	327	400
BZ03	339	297	216	298	272	-	306	153	178	327	400
BZ04	339	306	221	298	272	197	306	153	178	327	400
BZ05	339	297	216	-	272	-	306	153	178	327	400
BZ06	339	297	216	298	272	197	306	153	178	327	400
BZ07	339	306	221	-	272	-	306	153	178	327	400
BZ08	339	306	221	289 *	272	188 *	306	153	178	327	400
BZ09	339	306	221	-	272	-	306	153	178	325	400
BZ10	339	297	221	298	272	197	306	153	178	327	400
BZ11	339	306	221	-	272	-	306	153	178	327	400

* BZ08 Ft-M5 and Ft-M10 hit is not 100% length. -: The size could not be determined.

**Table A7:** Differences between size (Table A5) and genomic size (Table A6) of PCR products.

ID	Ft-M2	Ft-M3	Ft-M4	Ft-M6	Ft-M20A	Ft-M20B	Ft-M22	Ft-M23	Ft-M24
BZ01	0.8	6.6	2.9	3.7	1.7	−2	−3.3	0.4	0.3
BZ02	0.7	6.4	3	2.9	1.7	−1.2	−3.3	0.4	−0.3
BZ03	0.5	6.2	2.8	4	1.7	−1.3	−3.3	0.4	0.3
BZ04	1	7.1	3.4	3.8	1.7	−1.5	−3.3	0.4	0.1
BZ05	0.7	6.5	2.8	3.9	1.7	−1.5	−3.3	0.6	0
BZ06	0.8	6.6	2.8	3.7	1.7	−1.4	−3.3	0.5	0.1
BZ07	1	7.4	3.5	4.1	1.7	−1.4	−3.3	0.6	0.1
BZ08	0.8	7.1	4.6	3.8	1.7	−1.4	−3.3	0.6	−0.3
BZ09	0.8	7	4.3	3.8	1.7	−1.4	−3.3	2.3	0.2
BZ10	1	6.7	3.3	3.7	1.7	−1.5	−3.3	0.3	0.2
BZ11	1	7.5	3.3	3.8	1.7	−1.4	−3.3	0.3	0.3
Median diff.	0.8	6.7	3.3	3.8	1.7	−1.4	−3.3	0.4	0.1
Average diff.	0.8	6.8	3.3	3.7	1.7	−1.5	−3.3	0.6	0.1
Repeat size	6	9	5	21	12	12	6	23	21
